# A dynamic model to predict long-term outcomes in patients with prolonged disorders of consciousness

**DOI:** 10.18632/aging.203840

**Published:** 2022-01-19

**Authors:** Junwei Kang, Lianghua Huang, Yunliang Tang, Gengfa Chen, Wen Ye, Jun Wang, Zhen Feng

**Affiliations:** 1 Department of Rehabilitation Medicine, First Affiliated Hospital of Nanchang University, Nanchang 330006, Jiangxi, P.R. China

**Keywords:** prognosis, clinical prediction, disorders of consciousness, nomogram

## Abstract

Purpose: It is important to predict the prognosis of patients with prolonged disorders of consciousness (DOC). This study established and validated a nomogram and corresponding web-based calculator to predict outcomes for patients with prolonged DOC.

Methods: All data were obtained from the First Affiliated Hospital of Nanchang University and the Shangrao Hospital of Traditional Chinese Medicine. Predictive variables were identified by univariate and multiple logistic regression analyses. Receiver operating characteristic curves, calibration curves, and a decision curve analysis (DCA) were utilized to assess the predictive accuracy, discriminative ability, and clinical utility of the model, respectively.

Results: Independent prognostic factors, such as age, Glasgow coma scale score, state of consciousness, and brainstem auditory-evoked potential grade were integrated into a nomogram. The model demonstrated good discrimination in the training and validation cohorts, with area-under-the-curve values of 0.815 (95% confidence interval [CI]: 0.748–0.882) and 0.805 (95% CI: 0.727–0.883), respectively. The calibration plots and DCA demonstrated good model performance and clear clinical benefits in both cohorts.

Conclusions: Based on our nomogram, we developed an effective, simple, and accurate model of a web-based calculator that may help individualize healthcare decision-making. Further research is warranted to optimize the system and update the predictors.

## INTRODUCTION

Prolonged disorders of consciousness (DOC) caused by severe brain injury or nervous system dysfunction have attracted great attention in the neuroscience community. Prolonged DOC are defined by a coma lasting for > 28 days after severe craniocerebral injury [1]. Depending on the type of DOC after severe brain injury, patients may be in minimally conscious (MCS) or persistent vegetative (VS) states [2, 3]. Approximately 100,000–300,000 patients have been diagnosed with prolonged DOC in the United States [4], and the prevalence ranges from 0.2 to 6.1 patients per 100,000 people in Europe [5]. No accurate data is available for China, but the incidence and prevalence of prolonged DOC are believed to have increased progressively there.

The treatment options for prolonged DOC, such as hyperbaric oxygen, drug therapy, and nerve electrical stimulation therapy, have been studied for many years; however, no single accurate and effective treatment has been identified. Patients with prolonged DOC need long-term medical care and nursing, which brings a great burden to their families and society. The lifelong medical cost of a patient with prolonged DOC can be as high as US $1 million [6]. Therefore, it is particularly important to develop a simple and practical prognostic prediction tool that can help physicians make clinical decisions.

At present, there are a few prognostic prediction models for prolonged DOC. A number of studies have explored the relationship between neuroimaging and biomarkers and the prognosis of patients with prolonged DOC. Ming et al. predicted the 1-year outcome of patients with prolonged DOC using resting-state cerebral functional magnetic resonance imaging [7]. Xi et al. demonstrated that sleep electroencephalogram (EEG) structures were related to short-term prognosis of patients with prolonged DOC [8]. Taylor et al. utilized three biomarkers, GFAP, UCH-L1, and MAP-2, to predict the recovery of patients with prolonged DOC within a 6-month period [9]. Although considerable effort has been made to prognosticate prolonged DOC, the above investigative modalities are difficult to obtain and relatively expensive, which limits their widespread clinical application. In addition, existing research only examined the short-term (6–12 months) prognosis of prolonged DOC. There are no long-term prognostic prediction models for prolonged DOC. Thus, this study aimed to develop a simple, practical, and accurate clinical prediction model to prognosticate the 3-year outcomes for patients with prolonged DOC.

## RESULTS

### Baseline patient characteristics

A total of 151 patients from the Department of Rehabilitation Medicine of the First Affiliated Hospital of Nanchang University served as the training cohort, whereas 134 patients from the Shangrao Hospital of Traditional Chinese Medicine served as the validation cohort. The general data of the training and validation cohorts are shown in Table 1. During the follow-up period, 54 and 97 people in the training cohort had good and adverse outcomes, respectively. The poor prognosis rate was 64.2%. In the validation cohort, 57 and 77 patients had good and adverse outcomes, respectively, and the poor prognosis rate was 57.5%.

**Table 1:** Baseline characteristics of the training set and validation set.

**Features**	**Training set (n=151)**	**Validation set (n=134)**	**P-value**
Age(years)	48.88±14.46	53.22±14.52	0.012
Sex			0.104
Male	103(68.2%)	78(58.2%)	
Female	48(31.8%)	56(41.8%)	
Etiology			0.192
Trauma	74(49.0%)	75(56.0%)	
Stroke	67(44.4%)	46(34.3%)	
Anoxia	10(6.6%)	13(9.7%)	
CRS-R total score	5.00(3.00, 8.00)	5.00(2.00, 8.00)	0.771
GCS total score	9.00(7.00, 9.00)	8.00(6.00, 9.00)	0.208
Serum albumin(g/L)	37.48±4.14	36.67±4.45	0.111
Hemoglobin(g/L)	113.11±15.83	107.69±15.42	0.004
Basic cardiopulmonary diseases			0.521
Presence	18(11.9%)	12(9.0%)	
Absence	133(88.1%)	122(91.0%)	
Level of consciousness			0.604
VS	97(64.2%)	90(67.2%)	
MCS	54(35.8%)	44(32.8%)	
Multiple injuries			0.854
Presence	38(25.2%)	35(26.1%)	
Absence	113(74.8%)	99(73.9%)	
EEG background activity			0.288
Lack of alpha rhythms	75(49.7%)	75(56%)	
Alpha rhythms exists	76(50.3%)	59(44%)	
N20 on SEP			0.841
Presence	122(80.8%)	107(79.9%)	
One or both absent	29(19.2%)	27(20.1%)	
BAEP grade			0.006
GradeI-II	82(54.3%)	51(38.1%)	
GradeIII-IV	69(45.7%)	83(61.9%)	
Midline shift			0.848
Presence	18(11.9%)	15(11.2%)	
Absence	133(88.1%)	119(88.8%)	
Hypertension			0.724
Presence	48(31.8%)	40(29.9%)	
Absence	103(68.2%)	94(70.1%)	
Smoking history			0.718
Presence	19(12.6%)	15(11.2%)	
Absence	132(87.4%)	119(88.8%)	
Cholesterol			0.203
>5.17mmol/L	16(10.6%)	21(15.7%)	
≤5.17mmol/L	135(89.4%)	113(84.3%)	
Triglyceride			0.747
>1.70mmol/L	48(31.8%)	45(33.6%)	
≤1.70mmol/L	103(68.2%)	89(66.4%)	
Outcome			0.242
Good outcomes	54(35.8%)	57(42.5%)	
Adverse outcomes	97(64.2%)	77(57.5%)	

### Predictive variable screening

Univariate analysis demonstrated that age, Glasgow coma scale (GCS) score, Coma recovery scale-revised (CRS-R) score, state of consciousness, EEG background activity, N20 on somatosensory evoked potentials (SSEP), and brainstem auditory evoked potential (BAEP) grade were correlated with the prognosis of prolonged DOC. Multivariate logistic regression analysis demonstrated that four of these variables, particularly age, GCS score, state of consciousness, and BAEP grade, were independent prognostic factors for prolonged DOC (Table 2).

**Table 2:** Univariate and multivariate logistic regression analyses of prognostic factors in patients with prolonged DOC in training set.

**Variable**	**Univariate**		**Multivariate**	
	**OR(95%CI)**	**P-value**	**OR(95%CI)**	**P-value**
Age(years)	1.041(1.016-1.069)	0.002	1.037(1.006-1.071)	0.022
Sex				
Female	Ref			
Male	0.978(0.478-2.001)	0.952		
Etiology				
Trauma	1.099(0.552-2.186)	0.788		
Stroke	Ref			
Anoxia	1.389(0.329-5.864)	0.654		
CRS-R total score	0.834(0.748-0.923)	0.006	1.073(0.895-1.294)	0.399
GCS total score	0.612(0.473-0.768)	0.005	0.699(0.499-0.947)	0.027
Serum albumin(g/L)	0.921(0.844-1.001)	0.560		
Hemoglobin(g/L)	0.990(0.969-1.011)	0.356		
Basic cardiopulmonary diseases				
Presence	Ref			
Absence	0.517(0.160-1.672)	0.264		
Level of consciousness				
VS	Ref		Ref	
MCS	0.202(0.096-0.410)	0.000	0.309(0.087-1.039)	0.043
Multiple injuries				
Presence	Ref			
Absence	1.237(0.580-2.639)	0.581		
EEG background activity				
Alpha rhythms exists	Ref		Ref	
Lack of alpha rhythms	4.216(2.047-8.686)	0.000	2.252(0.958-5.428)	0.065
N20 on SEP				
Presence	Ref		Ref	
One or both absent	10.02(2.282-44.075)	0.000	3.24(0.712-23.580)	0.168
BAEP grade				
GradeI-II	Ref		Ref	
GradeIII-IV	4.987(2.395-11.010)	0.000	2.779(1.150-7.024)	0.026
Midline shift				
Presence	Ref			
Absence	0.474(0.148-1.521)	0.202		
Hypertension				
Presence	Ref			
Absence	0.978(0.478-2.001)	0.952		
Smoking history				
Presence	Ref			
Absence	0.605(0.205-1.782)	0.358		
Cholesterol				
>5.17mmol/L	Ref			
≤5.17mmol/L	0.798(0.262-2.430)	0.690		
Triglyceride				
>1.70mmol/L	Ref			
≤1.70mmol/L	1.650(0.816-3.338)	0.162		

### Development of the nomogram

**Figure 1:**
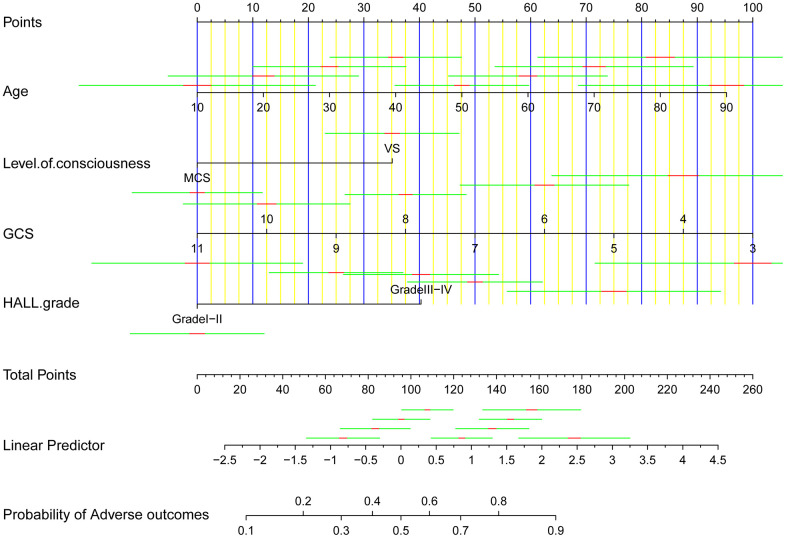
A clinical feature model was used to develop a nomogram.

Based on the results of the logistic regression analysis, four predictive variables (age, GCS score, state of consciousness, and BAEP grade) and an outcome variable (Glasgow outcome scale [GOS]) were utilized to construct a 3-year outcome prediction nomogram for patients with prolonged DOC (Figure 1). The sum of the scores for each predictive variable were determined. The higher the total score, the higher the probability of adverse outcomes.

### Establishment of a web-based calculator

**Figure 2:**
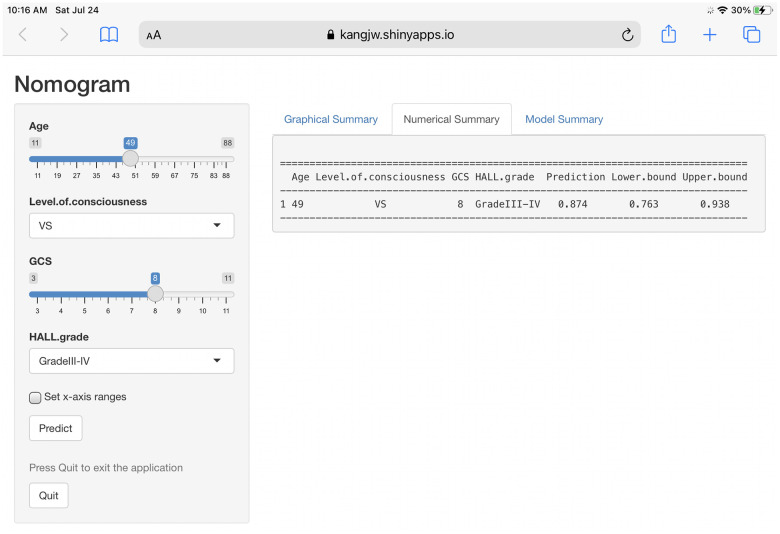
Construction of a web-based calculator for predicting outcomes of prolonged disorders of consciousness based on the model (https://kangjw.shinyapps.io/dynnomapp).

To apply our findings in the clinical setting, we developed a web-based calculator (https://kangjw.shinyapps.io/dynnomapp) to predict the 3-year outcomes for patients with prolonged DOC (Figure 2). For example, a 49-year-old patient in a VS with a GCS score of 8 and BAEP grade of 3 had an approximately 87.4% (95% confidence interval [CI] 76.3–93.8) probability of a poor prognosis within 3 years.

### Model performance in the training set

**Figure 3:**
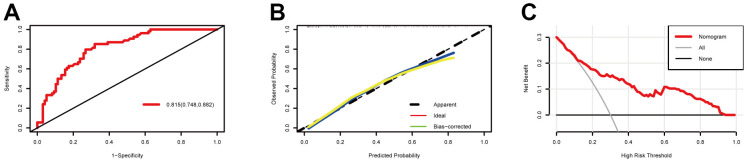
**Model discrimination and performance in the training set.** (**A**) Receiver operating characteristic curves for nomogram-based prognostic prediction. (**B**) Calibration plot examining estimation accuracy. (**C**) Decision curve analyses assessing clinical utility.

We evaluated the prediction model with our training cohort. Discrimination refers to the ability of the model to correctly distinguish between non-events and events and is evaluated by the area-under-the-curve (AUC). As demonstrated in Figure 3A, the AUC value of the nomogram in the training cohort was 0.815 (95% CI: 0.748–0.882), which indicated that the model had good discrimination. In contrast, the degree of calibration measures the numerical agreement between the predicted probability and the actual results. The calibration plots of the training cohort in this study demonstrated good consistency between nomogram prediction and actual observation (Figure 3B).

A decision curve analysis (DCA) is considered suitable for evaluating alternative prognostic strategies and has advantages over other commonly used measures [10]. For this study, we used the DCA to evaluate the clinical usefulness of our prognostic nomogram, and as demonstrated in Figure 3C, given a >10% probability threshold, patients with prolonged DOC gained more from our prognostic nomogram than from hypothetical treat-all or treat-none scenarios.

### Model performance in the validation set

**Figure 4:**
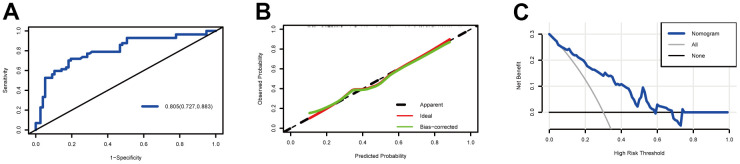
**Model discrimination and performance in the validation set.** (**A**) Receiver operating characteristic curves for nomogram-based prognostic prediction. (**B**) Calibration plot examining the estimation accuracy. (**C**) Decision curve analyses assessing clinical utility.

We evaluated the prediction model with our validation cohort. We utilized a receiver operating characteristic (ROC) curve, calibration curve, and DCA to evaluate the discriminative ability, calibration ability, and clinical effectiveness, respectively, of our nomogram. As shown in Figure 4A, the AUC value in our validation cohort was 0.805 (95% CI: 0.727–0.883), which indicated that the nomogram had good discriminative power in predicting the prognosis. The calibration curve for our validation cohort was close to the diagonal, which indicated a high calibration ability (Figure 4B). Within a large threshold probability range, the DCA curve was far from the extreme value (Figure 4C), which indicated that our model had more net benefits in predicting the prognosis of patients with prolonged DOC.

## DISCUSSION

Prolonged DOC has a wide range of clinical outcomes, and the diagnosis and treatment of prolonged DOC has become a worldwide concern. More accurate and practical prediction models are needed to help physicians construct treatment plans. Currently, only a few prognostic prediction models are available for patients with prolonged DOC, and almost all the available models only predict short-term outcomes [11]. To the best of our knowledge, this is the first study that developed a nomogram and web-based calculator to predict 3-year outcomes for patients with prolonged DOC. The present model was established on several readily available variables, based on four features that included age, GCS score, state of consciousness, and BAEP grade; and demonstrated superior predictive power in the training and validation cohorts. The AUC values of the nomogram in the training and validation cohorts were 0.815 and 0.805, respectively. In addition, we utilized calibration curves and the DCA to evaluate the calibration and clinical effectiveness of the model in two data sets. Our results suggested that the model was cost-effective for predicting the prognosis of patients with prolonged DOC as well as for assisting clinical decision-making.

The prognosis of prolonged DOC is affected by many factors, and as such, predicting the functional outcomes of prolonged DOC is very challenging. Research has demonstrated that the prognostic predictions in prolonged DOC should be based on a variety of variables in order to optimize accuracy [1]. When a single variable is used to predict prognosis, the risk for a wrong prediction is high. The neuroscience community is currently exploring new prognostic predictors for prolonged DOC, and researchers currently favor the use of functional magnetic resonance imaging, positron emission tomography, EEG, and other emerging brain monitoring technologies [11–13]. However, these examination methods are expensive and difficult to apply in the clinical setting; moreover, there are no available multivariate prediction models.

Our model utilized easily obtainable variables that are easy to apply in clinical practice. Age was one such variable that was an important predictor in our model. Previous studies have demonstrated an association between age and the prognosis of prolonged DOC, but there seems to be a stronger correlation between age and long-term prognosis than between age and short-term prognosis, with poor prognosis more likely in elderly patients as their resistance to disease progression is significantly weaker than in younger patients.

Previous studies have shown that patients with non-traumatic brain injury (TBI)-induced DOC lasting more than 3 months and TBI-induced DOC lasting more than 12 months are less likely to regain consciousness [14]. Most of the existing studies on patients with DOC examined prognosis between 14 days and 6 months [15], and only a few studies have examined patients over a 12-month time period from onset [1]. Existing studies have also demonstrated that some patients with DOC lasting more than 12 months do regain consciousness [16–18]; in particular, patients with MCS have been shown to regain the ability to live independently at home [19, 20]. Our study confirmed the above findings, and during the 3-year follow-up, patients in MCSs demonstrated better prognoses than patients in VSs.

In the past two decades, the GCS score has become the worldwide standard to describe levels of consciousness, and it is now one of the most widely used tools for assessing DOC in clinical practice [15, 20]. Our study demonstrated an obvious correlation between the GCS score and patient prognosis, which was consistent with that in previous reports.

First proposed by Greenberg in 1977 [21], the BAEP test is considered a good clinical detection index [22] that is highly accurate in predicting the prognosis of DOC, and is not easily affected by sedative drugs. Higher grades in the Hall classification correlate with worse prognosis [23]. In this study, our data demonstrated that patients with poor waveform differentiation (grades III and IV) had worse outcomes than patients with good waveform differentiation (grades I and II).

This study developed a simple and accurate model with the four clinical parameters discussed above. While previous studies have also shown that N20 in the SSEP and CRS-R scores can predict the prognosis of DOC [24, 25], these were not utilized as predictive variables in this study, which may be related to its heterogeneity.

Compared with previous studies, our study has several advantages. First, the nomogram is a useful model, in that it can integrate regression results, provide graphic and visual data, and predict individual risk in a highly detailed and intuitive manner. Second, previous studies predicted short-term outcomes, whereas this study utilized outcome variables observed over a 3-year period to build a prediction model for prolonged DOC. Our method provides a solid foundation for future clinical decision-making. In addition, this study used clinically accessible variables for joint prediction and externally verified the constructed model. The model demonstrated high accuracy and clinical use.

This study has some limitations. First, this was a retrospective cohort study with a small sample size that examined patients with complete outcome indicators. As such, it was prone to both information and selection bias, which are also the inherent limitations of retrospective studies. Large, prospective cohort studies with well-designed and standardized implementation protocols are required to verify our findings. Second, our population was heterogenous, and the majority of our patients did not have data on other important predictors, such as N20 and CRS-R scores, so these predictors were not included in our nomogram. Third, our study population was exclusively Chinese, which may limit the ability to apply its findings to a wider population.

In conclusion, our study identified age, GCS score, state of consciousness, and BAEP grade as important prognostic indicators of prolonged DOC after we developed a novel nomogram and web-based calculator based to predict the 3-year outcomes for patients with prolonged DOC. These results may help improve clinical decision-making and individualize treatment for patients with prolonged DOC.

## MATERIALS AND METHODS

### Subjects

This study was approved by the ethics committee (2020-061-3) of the relevant institutions. The patient data analyzed in this study were collected from the medical record information systems of two centers, namely the First Affiliated Hospital of the Nanchang University and the Shangrao Hospital of Traditional Chinese Medicine. The keywords "coma" and "disorders of consciousness" were entered into the database, and all patients admitted between June 1, 2015 and June 1, 2018 were included.

Patients with prolonged DOC of at least 28 days (where the illness is relatively stable and meets the internationally recognized diagnostic criteria for prolonged DOC) [1], a clinical diagnosis of VS or MCS, and in whom anoxic, traumatic, or vascular (i.e., hemorrhagic or ischemic) etiologies were identified were included in this study.

Patients who had a previous history of craniocerebral injury or DOC not caused by craniocerebral injury, who were unable to follow up, or who had incomplete outcome variables (i.e., GOS score) [26] or medical records were excluded from this study.

A total of 151 patients from the Department of Rehabilitation Medicine of the First Affiliated Hospital of Nanchang University served as the training cohort, whereas 134 patients from the Shangrao Hospital of Traditional Chinese medicine served as the validation cohort.

### Clinical data collection

A self-designed questionnaire was utilized to collect clinical data. GOS scores were recorded in the follow-up records and obtained from the patients’ family members through telephone consultation; for which the data collectors underwent formal training.

Eighteen potential predictive variables, which included baseline demographic data (age, sex, and etiology as trauma, stroke, or anoxia), patient condition at admission (state of consciousness as VS or MCS, GCS score, and CRS-R score), laboratory examination results (albumin, hemoglobin, cholesterol, and triglyceride levels, midline displacement on cranial computed tomography, EEG background activity, N20 on SSEP, and BAEP grade), and medical history (hypertension, smoking history, multiple injuries, and basic cardiopulmonary diseases) were evaluated.

The neuroelectrophysiological examination classified EEG background activity into five categories of severity according to recently proposed criteria for patients with DOC [27]: (1) normal EEG activity, characterized by predominant posterior alpha rhythm with an anterior-posterior gradient (APG), and without focal or hemispheric slowing or epileptiform abnormalities; (2) mildly abnormal (MiA) EEG, characterized by predominant posterior theta activity (>20μV), symmetric or not, with frequent (10–49% of recording) posterior alpha rhythms; (3) moderately abnormal (MoA) EEG, characterized by predominant posterior theta activity (>20μV), symmetric or not, poorly organized APG, rare (<1% of recording) or occasional (1–9% of recording) posterior alpha rhythms; (4) diffuse slowing (DS), defined as EEG background activity with predominant diffuse theta or theta/delta rhythms with amplitude >20 μV, without APG; and (5) low voltage (LV) EEG, defined as predominant EEG activity (theta or delta) <20 μV over most brain regions. According to the latest literature reports, this study categorized patients based on the presence of posterior alpha (normal, Mia, MOA) or lack of posterior alpha (DS, LV) rhythms [28].

This study also utilized the Hall classification for BAEP grading [23]: Grade 1, normal; Grade 2, slightly abnormal, with moderate waveform differentiation, and with the following possible problems: prolonged peak latency of the I, III, or (and) V waves, prolonged interpeak latency of the I–III, III–V, or (and) I–V waves, peak-to-peak latency ratio of III–V/I –III >1, and amplitude ratio of V/I <0.15; Grade 3, moderate abnormality, poor waveform differentiation and repeatability, with the following possible problems: prolonged peak latency of the III or V waves, disappearance of the V wave; and Grade 4, severe abnormality, only the I wave exists or disappearance of all waveforms. This study divided the BAEP grades according to the degree of waveform differentiation into the good (grades 1 and 2) and poor (grades 3 and 4).

SSEP readings were divided according to the presence or absence of N20 (unilateral or bilateral absence). Functional outcomes were assessed at follow-up using the GOS [26], which included five categories: 1, death; 2, vegetative state (no response to instructions); 3, severe disability (unable to take care of themselves but can follow instructions); 4, moderate disability (able to self-care but unable to return to work or study); and 5, good recovery (able to return to work or study).

For the purpose of analysis and based on established literature [29], outcomes were categorized as good (moderate disability or good recovery) and bad (death, vegetative state, or severe disability).

### Statistical analysis

All potential predictors were analyzed using univariate analysis. Indices with statistical significance were further analyzed using logistic multivariate regression analysis, which identified the predictive variables that were integrated into the nomogram. To apply our findings in the clinical setting, we developed a web-based calculator with computer programming technology. We evaluated the final model in the training and validation cohorts with the ROC curve, calibration curve, and DCA. All analyses were performed using the software R, version 3.6.2 (The R Project for Statistical Computing, Vienna, Austria). A *p*-value <0.05 was considered statistically significant.
